# Interpersonal Relations Within the Context of Resource Groups for People With Severe Mental Illness: A Narrative Approach

**DOI:** 10.3389/fpsyt.2021.632437

**Published:** 2021-02-12

**Authors:** Cathelijn D. Tjaden, Jenny Boumans, Cornelis L. Mulder, Hans Kroon

**Affiliations:** ^1^Department of Reintegration and Community Care, Trimbos Institute, Utrecht, Netherlands; ^2^Department of Social and Behavioral Sciences, Tranzo Scientific Center for Care and Welfare, Tilburg University, Tilburg, Netherlands; ^3^Department of Psychiatry, Erasmus Medical Center, Rotterdam, Netherlands; ^4^Research Department ESPRi, Parnassia Psychiatric Institute, Rotterdam, Netherlands

**Keywords:** recovery, family involvement, empowerment, resource group, severe mental illness, assertive community treatment, narrative analysis, interpersonal dynamics

## Abstract

**Objective:** The resource group method intends to promote patients' agency and self-management and to organize meaningful partnerships between patients and their informal and formal support systems. The aim of this study was to enhance the understanding of interpersonal dynamics that arise within resource groups for people with severe mental illness. Insight into these unfolding processes would enable improved implementation of the resource group method so that it contributes to establishing a positive social environment, which can lead to more enduring recovery.

**Methodology:** We performed a narrative analysis of transcripts and field notes obtained in a longitudinal, qualitative study on the resource group method. The stories of four different resource groups were reconstructed and analyzed in depth. Data included a total of 36 interviews (with patients, significant others, and mental health professionals) and 18 observations of resource group meetings.

**Results:** The degree to which the resource group method actually contributes to recovery was based on the extent to which the existing roles of and patterns between the patient and his/her resource group members were altered. Breaking through old patterns of inequality and the joint search for a new balance in relationships proved to be crucial processes for establishing an empowering resource group. The four cases showed that it takes time, patience, and small steps back and forth to overcome the struggles and fears related to finding new ways of relating to each other. An honest and reflective atmosphere in which all participants are encouraged to participate and be curious about themselves and each other is essential for changes in interpersonal dynamics to emerge. Such changes pave the way for individuals with SMI to find their own voices and pursue their unique recovery journeys.

**Conclusions:** The functioning of the resource group and the ability of the involved members to respond in new ways are important when working toward the patient's recovery goals. The resource group method should therefore not be considered an intervention to organize informal support for the patient, but a platform to expose and adjust the functioning of the patient's social network as a whole.

## Introduction

Over the past 40 years, a confluence of factors has contributed to the evolution of a renewed view of mental health recovery for people with severe mental illness (SMI). There is increased recognition that patients are surrounded by social networks that may support, undermine, substitute, or supplement professional help (1, 2). Together with processes of deinstitutionalization and changing ideas about “good care,” this recognition has led to an increased focus on community care in the last few decades (3, 4). Simultaneously, the consumer/survivor movement has fought for patients' right to co-decide and co-create the care and support they receive, and it has aimed to achieve greater empowerment for patients, de-stigmatization, and renewed hope for the future (5, 6). As a consequence, international policies and guidelines now emphasize the importance of partnerships between mental health professionals, service users, and their social networks to improve service quality and enhance the empowerment and involvement of service users and their significant others (7).

Evolving from this movement, the resource group (RG) method (8) is a promising way to combine the call for agency and self-management with the appeal to organize meaningful partnerships and establish care that is embedded within community life. The origins of the RG method lie in the Optimal Treatment (OT) model, which integrates biomedical, psychological, and social strategies in the management of SMI (9, 10). In Sweden, the model was further developed and relabeled as Resource Group Assertive Community Treatment (R-ACT) (11–13), in which ACT teams were enriched by RGs. Research on R-ACT has focused on effectiveness and found improvements in functioning, well-being, and symptoms for people with psychosis (12, 13). Implementation and effectiveness of RGs outside Sweden is being investigated (14).

To create an RG, patients invite significant others from their informal network (such as friends and family) and their formal network (such as mental health nurses, social workers, or job coaches). Each RG has a unique composition that is suited to the individual and their recovery wishes and needs. During RG meetings, which are held quarterly, the RG discusses the patient's goals and wishes and jointly determines a recovery plan (8).

Central to the RG method is the assumption that recovery emerges from the relationship between individuals and the social and cultural environments in which they are embedded (15–17). Extensive research indicates that the presence and involvement of significant others contributes to recovery, as they are a source of warmth, support, and encouragement. For example, family members possess a deep knowledge of the patient from years of “standing alongside the person,” and can prevent them from adopting a stigmatized, illness-related self-image (18). Also, families can encourage engagement with treatment plans and recognize early warning signs of relapse (19), and they can assist the patient in accessing services during periods of crisis (20–22). In addition, it has been reported that families can provide practical assistance, such as by offering temporary housing or cooking meals (23).

However, establishing positive social support and rebuilding beneficial social networks that enable recovery are recognized as challenging features of treatment programs. Some forms of assistance or specific behaviors or communications can unintentionally lead to aversive events or stress for the person with SMI. Thorough investigations have found that high levels of expressed emotions within the social environment—referring to close kin's criticism, hostility, and over-involvement in relation to a relative with schizophrenia—can be a source of stress that negatively impacts the course of the psychiatric disorder (24, 25). In addition, the involvement of significant others can impede the recovery process when they remain fixated on a helper role and are unable to support an individual's movement toward autonomy and reciprocal relationships (23, 26). Also, family members who do not understand how environmental cues, adverse events, or stress can increase the risk of relapse might act in ways that increase risks without realizing it (27).

Taken together, although the involvement of significant others in treatment and care has been broadly acknowledged as a source of support that leads to more positive outcomes, more knowledge about interpersonal dynamics is needed to shape social support interventions. In addition, while mental health professionals fulfill an important part of the interpersonal dynamics within a RG, beneficial and hindering aspects of their attitudes are not well-understood. Hence, the aim of this paper is to provide an in-depth understanding of the interpersonal dynamics that arise within RGs and their influence on the recovery journey of the individual suffering from SMI and his/her significant others. Insight into these unfolding processes enables improved implementation of the RG method so that it contributes to establishing a positive social environment, which leads to a more enduring recovery for people with SMI.

## Methodology

### Resource Group Method

To work according to the RG method (11–14, 28), the patient first asks his/her significant others and mental health professionals to join the RG. This is referred to as *nominating*. Then, the patient is stimulated to take the lead in preparing the first RG meeting by deciding on the location and chairman (preferably the patient themself). In addition, together with a mental health professional, they develop an RG plan that contains the recovery goals they want to discuss during the meeting. Before the first meeting, the professional separately invites all nominated RG members to engage in an in-depth preparatory conversation to discuss the relationships among the nominee, the patient, and the other RG members as well as the role the nominee wants to have in the RG. Follow-up RG meetings are scheduled, on average, once every 3 months. The composition of the RG is flexible and might change over time depending on the patient's goals, wishes, and phase of recovery. In the present study, the RG method was implemented in the context of Flexible Assertive Community Treatment (FACT) (29), the most frequently used outreach service in the Netherlands. FACT involves a multidisciplinary team who provides individual care—including case management and home visits—and scales up to team care with intensive, full ACT when needed.

### Design

This paper is based on a narrative phenomenological-hermeneutic analysis [(30), p. 295] of transcripts and field notes that were derived as part of a larger qualitative study exploring multiple perspectives on the RG method for people with SMI. The methodology of the larger study, including the recruitment of the cases, is described in depth elsewhere (31). In short, the study used a longitudinal multiple case-study design based on grounded theory (32, 33) to explore the developments and processes in eight RGs. Five of these cases were studied by the first author, and three were studied by the second author. In the current paper, the five cases studied by the first author are re-analyzed from a within-case perspective. One case was dropped because no informal network was involved in the RG and thus it contained too little information on the topic of interest: interpersonal processes within RGs. Hence, four cases were analyzed in the current paper.

### Data Collection

Data were collected between November 2017 and December 2019. Data collection for each case started when the RG was set up. Data was collected through four means. First, a narrative interview was conducted with the patient at the start of data collection based on a global topic list (34) (see Supplementary Material). Second, the RG meetings were observed and audio-recorded. Third, between the meetings, repeated in-depth interviews were conducted with the patients about their daily life, perceptions of their goals and aspirations, relations with the social environment, and experiences with the RG. Later in the process, the initial interpretations of the data were discussed with the patients during these interviews. Fourth, by the end of data collection, all the RG members (patients, significant others, and professionals) were interviewed about their experiences with the RG. Throughout the study period, a personal connection was established between the researcher and patient via telephone calls and messages.

The interviews with the patients were interactive and guided by neutral, open questions. Participants were encouraged to discuss topics that they considered relevant. Hereby, these interviews were aimed to co-construct understanding of the meaning and unfolding of the RG (35–38). The interviews took place at the patient's home or another preferred location. There was no time limit, and the duration ranged from 20 min to 2 h. The interviews with RG members were somewhat more structured. The topics of those interviews were pre-determined by a topic list (see Supplementary Material), which was constructed by the first and second author based on the emerging themes and categories. Most interviews and RG meetings were recorded and transcribed verbatim. One participant (“Martin”) was difficult to reach, and most contact was informal and by phone. These contacts were not recorded and transcribed; instead, the researcher wrote field notes about the topics that were discussed.

Short field notes were written after every contact, interview, or RG meeting to describe the initial associations of the researcher. Cases were followed until within-case saturation occurred (i.e., the moment when new data collection no longer seemed to bring up major new developments in that particular case (39). Within-case saturation was defined based on the general research question of the larger qualitative study exploring multiple perspectives on the RG method for people with SMI, based on grounded theory. Consistent with the grounded theory approach of saturation categories (40), data collection continued until nothing new was being heard and all areas that seemed to warrant further investigation had been pursued. Hence when the first author observed that new data tend to be redundant of data already collected, and did not lead to new themes regarding the understanding of the role RG for that case, this was discussed with the second author in a meeting. When both agreed, the case was considered to be saturated. The time period to reach saturation ranged from 6 months to 2 years. For one of the cases (“Martin”), we had to stop data collection earlier, as he no longer answered his phone or called back. The first and second author were in constant dialogue during data collection to explore developments and discuss their interpretations. In the current paper, a total of 36 interviews (with patients, significant others, and mental health professionals) and 18 observations of RG meetings are analyzed. See Table 1 for an overview of the collected data for each participant.

**Table 1:** Overview of the collected data^b^, sorted per participant.

**Parti-cipant**	**Narrative interview**	**In between interviews**			**RG meeting**	**Evaluative interview**	**Interviews RG members informal**				**Interviews RG members formal**				**Total**
		**Visits**	**Phone**	**Total**			**Mother**	**Brother**	**Friend**	**Total**	**CM^a^**	**PS^a^**	**SW^a^**	**Total**	
John	1	4		4	7	1	1	1		2	1			1	16
Martin	1	2	2	4	1					0	3			3	9
Leon	1	4		4	6	1	1		2	3	1	1		2	17
Raoul		3		3	4	1	1	1		2	1		1	2	12
Total	3			15	18	3				7				8	54

a *CM, case manager; PS, peer support worker; SW, social work*.

b *As we lost contact with one of the participants during the data collection (Martin) we could not conduct the final interview, nor ask his RG members (n = 4) to participate. Also, one formal RG member (peer-worker John) did not respond to our request to interview them despite several attempts. Lastly, one of the participants (Raoul) had invited his mother and case-manager to the narrative interview, and therefore we couldn't follow the topic-guide. We added this interview to the “in-between” interviews*.

### Data Analysis

For the larger study (31), the first and second author had coded all transcripts and field notes together in an ongoing dialogue and had written memos of their discussions, so both were familiar with the data. For the present study, the first author reread the transcripts, field notes, and memos of the coding associated with a particular case several times, searching for excerpts that raised curiosity or questions related to the aim of the study. Puzzling parts of the data material could function as significant events and uncover possible plots (41, 42). After identifying the possibly significant events for each case, the first author constructed initial narratives for each case and thoroughly discussed them with the second author. Then, the first and second author read parts of the transcripts and the field notes again to search for possible explanations for the raised questions and for other parts of the data material that seemed connected to important developments. These data were used to reconstruct the narrative. The analysis followed the principles of the hermeneutic circle (30), which involves an interpretation process in which the research continuously goes back and forth between pieces of a text and the preliminary understanding of the whole narrative. This procedure continued until a satisfactory, coherent interpretation was achieved. This interpretation led to a deeper level of understanding of the experiences and interactions of all involved (43).

### Ethical Considerations

The Medical Ethical Committee of VU Medical Centre granted approval for the study (IDS: 2017.316). Written informed consent for publication and usage of anonymized quotes was obtained from all patients and informal RG members before data collection. We changed names and details to maintain participants' confidentiality.

### Reflexivity

The study is part of the PhD thesis of the first author, CT. Next to her work as a researcher, CT is a psychologist in an urban area with people facing diverse problems, both with regard to severity as well as nature. For her PhD, CT briefly followed the developments of 58 RG's throughout the country, although with an utterly different intensity compared to the four men of the present study. In addition, she was involved with the supervision of the mental health professionals implementing the method, including those involved with the four men. JB, the second author, is an experienced qualitative researcher with a focus on investigating and understanding service user's lived experiences with psychological suffering as well as the process of recovery. Before the start of the study, CT and JB took time to truly get to know each other and to share their personal stories to be able to promote each other's' reflexivity.

## Results

In the following section, we share the remarkably different stories of four men and their RGs: John, Leon, Martin, and Raoul. We narratively describe how their RGs developed over time and how the interpersonal relations evolved, both from their perspectives and from the perspectives of their significant others and professionals. The stories are not merely characterized by successes or smooth transitions toward meaningful collaboration and empowerment. They also reflect the struggles, the ups and downs, and the tensions that arise during a recovery journey. Above all, the stories provide insight into the unique and different ways in which the RG method takes shape in the lives of the four men. Each story ends with a short reflection by the researchers on the emerging interpersonal processes within that RG.

### Case 1: John—Agency vs. Dependency

At the start of data collection, John has just moved from the clinic—where he stayed after two severe psychotic episodes—to live with his brother. He sets up his RG with a peer worker and nominates his brother, mother, case manager, and social worker to be part of it. From the beginning, John is very involved in the RG method. He explains that he expects it to help him regain control and an active life now that he is out of the clinic. He enthusiastically appoints himself as chairman of the RG meetings, and he puts a lot of effort into making his RG plan and agenda. Together with living in a new city and being out of the clinic, he sees the beginning of the RG as a promising new start and aims to make some profound changes in life.

*RG meeting*. John: “It gives me a lot of space to think about stuff and to write things down myself. And I also think that the goal of the RG is to make sure that I get certain things done in my life, and that it can serve as a big stick when I postpone things or not keep my promises. That would be very nice. Because I have stood still for a few years and have not been doing anything at all and then it is obviously not going well.”

In the first few months of data collection, it becomes clear how deep John desires to get back to living a “normal life.” He feels challenged by the fast-moving world around him, in which everyone seems to be able to participate and to build a meaningful existence. His RG plan illustrates what a normal life would look like to him. It is filled with long-term, ambitious plans, varying from traveling the world to having a full-time paid job. He struggles to connect this with his current situation.

*Interview John*. John: “I have a lot of trouble to accept that I am being treated. Well…. Wait, I [said] it wrong. I have accepted it but I have a bit of trouble that I don't function fully as I used to.”

The RG increasingly becomes an audience to communicate his struggles. The researchers' field notes describe that John has a tendency to think thoroughly about everything, and that expressing himself in the RG meetings allows him to gain an overview of all the plans in his head and bring them closer to the world around him. In an in-between interview, John describes the RG meetings as a “platform” where he can share his thoughts and where he feels in control about decisions in his life. Although this is a positive experience for him, he clearly expresses that he is uncomfortable with actually asking for help from his family. During the meetings, he rejects their help and sometimes gets irritated when they try to advise him.

*RG meeting*. Peer worker: “And what can others in the group do about this?” John: “Well, I also said that I prefer to do as much as possible myself, that is really, really important to me. […].” Mother: “But don't you think it would be easier when your brother asks you, you know, ‘have you thought about this or that’. So that you keep your promises.” John: “no it will work out…. *sighs deeply. Mother wants to start a sentence but John interrupts her, talking fast* […]. I will keep my promises, I just have to be a bit more adequate. Faster, better understanding. I get it, I know how it goes. It will be alright.”

After about 6 months of data collection, John starts to drink again and is taking his medication irregularly, leading to several incidents. The nature of the RG meetings changes somewhat, and they evolve into a place where these incidents can be openly discussed. Not only the RG members but also John acknowledge the urgency of the situation, which paves the way for joint agreements. His mother and brother explain in interviews that the RG meetings provided an opportunity to make clear agreements on what to do in the case of an incident, and they appreciated the ability to quickly contact mental health professionals, especially because John has a tendency to downplay incidents. Importantly, the mutual trust is not violated, because John remains part of the conversation and gives his permission to discuss these difficult topics.

*RG meeting*. Mother: “So we have agreed that we can have a conversation with [case manager] about you, both your brother and I, if a crisis situation arises […].” John: “Yeah, that is when, if you have a signal. So when [brother] or you have that idea, and then you think that things are not going well again, then you immediately get in contact. And I'm just going to make sure it goes well.”

*Interview brother*. Brother: “It gives him confidence, I think, that one doesn't talk *about* him, but *with* him. Because if you don't do that, you'll get problems. Because in the past too much has been decided behind his back, and that made him very suspicious.”

In the period that follows, John starts to take classes and volunteers. He achieves more structure in his daily life, which he appreciates. Despite this, John doesn't follow up on the agreements made in previous RG meetings, and several incidents happen. Thus, the relationship between John and his family remains dominated by tension. Toward the end of data collection, the researcher's field notes state that although the RG has become a place for John to feel connected with the world around him, no actual *joint* recovery process arises. John still seems to interpret the help or involvement of others in his recovery journey as an infringement on his freedom and undermining of his agency. Most importantly, it conflicts with his idea of leading the “normal,” independent life that he desires. Both the mental health professionals and his family members look at it differently. In their final interviews, they claim that John's conception of agency is actually hindering his recovery, and that he has to learn to accept help from others to turn his ideas into actions suitable for his daily life. However, the RG meetings were not used to jointly reflect upon these differences in perception. According to the case manager, she was hesitant to facilitate a critical, open dialogue because there was a risk that John would be placed in a vulnerable position in relation to his family.

*Interview case manager*. Researcher: “Do you feel that he is more in control over his treatment?” *Silence*. Case manager: “No, I don't really think so. I think in his experience he is, also because he is the chairman and during that meeting he is really in that role. But I don't think he is more in control at this moment [in life]. In the sense that I, the mental health care professional, always have to get him to: what you are going to do now, what do you have to do, make sure that you pay attention to that, et cetera […].” Researcher: “So even though that—according to his words—the group gives him control, helps him to make decisions; that is not in line with the reality, with how it really goes?” Case manager: “Well, I'm afraid not. I think it is good that he has that feeling, but what is the value of it if I, and my colleagues, are still pretty tightly in charge of his functioning?”

#### Reflection

For John, the setting of the RG—in which he served as chairman and his significant others were there for him in the meetings—was encouraging, as from the very beginning it allowed him to experience agency and responsibility. The RG became a place in which John could feel socially connected with the world around him while being the one in charge. However, his own ideas about what he was able to do himself and what he needed others for did not quite match the perceptions of the people around him. John was very focused on not being a patient, and he could hardly tolerate talking about his vulnerabilities or accepting any help. In the interactions with his RG, the other members felt forced to emphasize the problems and risks in his life. As a result, John wanted even more to prove that he could be in charge and did not need others. By the end of the study, John's final goal remained doing everything independently, as he still perceived that as the ultimate form of agency. The RG members went along with this to prevent friction, although they believed that it was not in line with the current situation. Thereby, John and his RG were engaged in a vicious circle and seemed to be stuck in their roles. The difference in perceptions was not directly addressed in the RG meetings, and no openness or reflection emerged in communications. Thus, the RG as a whole was not encouraged to create a story that they all wanted to pursue, and the other members only partially believed in John and his efforts. John's experience of agency remained limited to the RG meeting and did not expand to his treatment, social relations or broader life.

### Case 2: Leon—Urged to Reshape Toward Reciprocity

Since early adolescence, Leon has been in contact with mental health care professionals. At the start of data collection, he has been in and out of different clinics for about 3 years, and he is looking for a way to find meaning in his daily life. He explains that his main struggle is regulating his emotions. In the past, he has experienced several blackouts with self-harming behaviors and overdosing on medication and drugs. During the first interview, Leon describes how insecure he feels about himself:

*Interview Leon*. Leon: “I still find it difficult to receive compliments or to hear positive things about myself. It is easier to identify myself with failure. I basically set the bar always too high for myself, so that I fail and it is confirmed that I am not worth it. […] That is one of the greatest core beliefs of my life. Like, I'm not worth it, I'm not worth anything.”

Leon is very motivated to work on himself and puts a lot of effort into fulfilling what is expected from him regarding the setup of the RG. In addition to his case manager, he nominates his partner, his mother, two friends, his music therapist, a peer worker on his FACT team, and a social worker from supported housing. Before and during the first RG meeting, Leon looks stressed. In the subsequent interview, he explains that he felt great pressure for it to be a good meeting. He found it difficult to believe that these people want to be there for him because they like him and care about him; instead, he feels like they are judging him:

*Interview Leon*. Researcher: “How do you feel when you're the chairman at the meeting?” Leon: “Very embarrassed. Embarrassed, a bit anxious. You know, have I prepared myself well enough, that kind of things just stick in my head all the time. It's just, yes, like if you take an exam, that feeling a little bit.”

The somewhat tense undertone of the first meeting persists in the following meetings. According to the researcher's field notes, although Leon easily shares his vulnerabilities and struggles, he does not talk about what he is truly thinking or feeling. He tends to inform the people around him after a difficult period but isolates himself in the moment, hesitant to ask for help because he fears putting strain on them. The members of his own network take a “wait and see” approach because—as they explain later—they don't really know what their role is and they are cautious to avoid stressing Leon even more. The professionals unintentionally reinforce this by mainly directing the conversation toward Leon and not so much toward his significant others. Thus, rather than serving as a strengthening, supportive atmosphere, the RG meetings emphasize Leon's vulnerable side and his role as the patient, and it is mostly the professionals and Leon making an effort to change the situation.

*Interview with friend*. Friend: “[…] the group was not being asked anything at all, like what do you want to do or what do you think we should do or something. Often, Leon was talking most of the time, and then the professionals said things, we will arrange a house for you, we will do medication, et cetera. And then nobody asked me, [other friend], or mother anything.”

About halfway through data collection, several important events take place that change the way the RG takes shape. After being his main source of support for many years, Leon's partner breaks up with him. In reaction, Leon is overwhelmed and feels severely depressed, not seeing any meaning in life. He experiences a blackout in which he overdoses and has to spend several nights on the intensive care. In the aftermath of this incident, frustration and difficulties arise regarding the communication between different parties (family, friends, and professionals). In the RG meeting that follows, an RG member—one of Leon's friends—asks for a joint evaluation. The RG then openly talks about the lessons learned, who can do what in case of an emerging incident, and how to improve communication in critical moments. This seems to be a first step toward the informal RG members' involvement as active and equal partners. A few weeks later, Leon again feels severely bad. The professionals actively stimulate him to get in touch with one of his friends and share how he feels in order to prevent another incident. When Leon does so, it becomes a positive and important experience for both Leon and his friend:

*RG meeting*. Friend: “I am glad that you contacted me during that period you felt so bad, and that you really told me what was going on inside you. Not only, well yes, I am feeling bad, but also why and what it did to you. It made me feel like I could better be there for you.”

From this experience, as he later comments in an interview, Leon learns that letting other people know what he truly feels and asking for help at difficult times is not a sign of weakness or dependence, but can be strengthening and rewarding, both for him and the other person. The atmosphere and content in the following RG meetings changes. The conversation is no longer solely directed toward Leon and his challenges; the RG members start to use the meetings as a platform to openly explore how *everyone* feels, reflect on the influence of their own behaviors, and discuss their thoughts and doubts. The open and reflective atmosphere that arises seems to function as a mirror for Leon, helping him to learn to express himself and his emotions. This allows him to start searching for his own voice, and gradually, he realizes that he is capable of being in charge of his own decisions:

*RG meeting*. Mother: “Yes, now you really choose […].” Leon: “[…] my own social contacts […]” Mother: “[…] things yourself. Just as well as deciding to grow your beard.” Leon *smiling shyly*: “Yes, that is indeed one of those choices.” Mother: “Yes. Your own choice.” *Silence*. Leon: “Little by little making my own choices. I definitely feel like I'm slowly growing in that […].” Case manager: “Yes, absolutely.”

At the time of the final interview, the researcher's field notes indicate that the RG has undergone a transformation process; the roles of the RG members have changed, and their mutual relationships have been gradually reshaped. In addition, Leon's use of language when speaking about his RG changes. While he first tended to use proto-professional phrases, such as “utilizing my support system” and “significant others,” he seems to have left those terms behind at the time of the final interview and replaced them with phrases such as “asking a friend to go for a beer and talk” when he is having a difficult time.

#### Reflection

An important development within this case was the break-up of Leon and his partner. When Leon could no longer rely on her, he was forced to find new ways to take care of himself. This new situation caused existing patterns and current relationships to come into question and be reshaped. Hence, the interaction pattern within the RG, in which Leon felt vulnerable and judged and his significant others were reserved and hesitant in order to spare his feelings, changed. The RG members slowly transformed from passive listeners into active participants. They started to reflect on themselves and the process, and they shared their needs, frustrations, and emotions. This stimulated Leon to also express himself. Thereby, the RG became reciprocal instead of unilateral in its functioning. Also, Leon started to believe that he was worth the attention of his RG and therefore could experience the RG as a source of support. He gradually moved beyond the role of patient and was able to take more charge in making decisions. As a result of these parallel and intertwined developments, the RG process became a joint effort and led to increased equality within mutual relationships. The case is a clear example of the fact that difficulties and tensions are unavoidable parts of a recovery journey, and jointly overcoming them may be key to moving in a fruitful direction.

### Case 3: Martin—Distance and Closeness

At the start of the first interview, Martin proudly shows a large grid drawn on the wall that represents the number of days he is clean from drugs. He is happy to finally be at a point in life where he could manage to take this step. However, being clean takes enormous strength, and he describes feeling constantly confused and tired. During the interview, Martin openly speaks about himself and the severe events that occurred in his young childhood. The past 10 years of his life have mainly revolved around his substance abuse and the associated lifestyle. He states that although he has been through a lot with his family, they are really close to him and he is grateful for their support. At the same time, he feels pressured by them, and he hopes the RG meetings will help him to be better understood. In addition to his case manager, he nominated his mother, stepfather, brother, sister-in-law, and coach from his volunteer work to be part of the RG.

*Interview Martin*. Researcher: “What do you hope [to achieve with the RG]?” Martin: “Well, uhm…. My parents and my brother have said a few months ago, yes, we now accept you the way you are, and if you relapse, well okay, you know. But now my mother tried to say the other day, why don't you try to work a bit more. And then I really said, mom, you shouldn't do that. You just have to let me do it my way, because if you are going to say that, then I immediately get more cravings, and the feeling that I am not accepted anymore. So I said, please, just let me do it at my own pace.”

The search for recognition and acceptance of his fight against addiction is a very important theme for Martin. In the preparation for the first RG meeting, he decides—with the help of his case-manager—to write a letter in which he reintroduces himself to his family and asks for some distance from them in order to recover. During the first RG meeting, he reads the letter out loud:

*RG meeting*. Martin: “Well here I am, and that is someone with an addiction and the associated lifestyle, that I am trying to get out of. That's a little bit how or who I am now. How it feels. My goal is to build a normal rhythm of life again, to be clean. To enjoy things again and to pick up my hobby again. […] At the moment I have mixed feelings, because despite the good feedback from everyone, I still feel that more is expected from me than is feasible at this moment, for example if I hold off the contact with you guys, from everything. But to stay clean requires so much energy, to alter the cravings to something else. […] From the inside, I feel really messed up at the moment, and that just demands all my energy now. So I need a bit of distance to be able to hang on.”

The letter and the way that Martin reads it impresses the family. They appreciate that he is honest, and they tell him that they understand his request for space. The RG jointly and respectfully talks about what everyone needs in this new situation. Later on in the meeting, when Martin shares his goals and wishes for the near future, the RG responds by expressing their positive beliefs and expectations. Martin afterwards comments that, despite the positive tone, their hopes and expectations made him feel pressured:

*Field notes*. “It had hurt him that his father had said that he actually wanted him to be like his little brother: work, girlfriend, house. He found that painful to hear, and he seemed to be annoyed about it too.”

In the period following the first RG meeting, the researcher and Martin have several informal contacts in which it is revealed that Martin is struggling to find the right balance between closeness and distance in both contacts with his family and the case manager:

*Field notes. “*Right after the RG meeting his brother stopped contacting him. Although this was what he had asked for, it made Martin feel upset, as he felt abandoned and not being part of the family. One month later, when the two brothers had talked about this and his brother had invited Martin a couple of times to come over, Martin felt pressured and unseen in how he feels because his brother was expecting too much.”

2 months after the RG meeting, it is revealed that Martin has used again and that he manipulated his mother to get money and his stepfather does not know about this. Martin expresses to the researcher that he feels deeply disappointed in himself. In the same period, several interpersonal tensions between members of his RG manifest: his stepfather threatens to reveal secrets about his mother to Martin and his brother, his sister-in-law and stepfather have a dispute and refuse to talk with each other, and the family is annoyed by the mental health professionals. Martin cancels the subsequent RG meeting. He explains that although he would like to continue in the long term, the idea of an RG meeting now causes him too much stress due to all the tensions. The last time the researcher gets in contact with Martin, he considers continuing the RG with a different composition because he wants to gain some distance from his family and focus on the future.

After a few months and several attempts, the researcher is no longer able to get in touch with Martin, and to respect this, she does not interview his family. About a year later, she hears from his case manager that Martin is setting up a new RG meeting with the same members.

#### Reflection

The RG meeting took a first step toward overcoming the existing interactional difficulties and working to (re)build mutual trust. However, both Martin and his family were entangled in a pattern of seeking distance and closeness. Therefore, Martin alternated between feeling pressured and abandoned. This complicated the establishment of satisfying interactions in which Martin's need to be truly seen and accepted could be acknowledged. When his family sought closeness and said they wanted the best for him, Martin felt as if he is only worth something when he is absent. This interpretation of conditional love and attention made him feel pressured to behave in a certain way. Drugs—and later distance—became a way to take back control and avoid being left and hurt. The interactional patterns of Martin and the other RG members seemed to be entangled with drug use, which made it difficult to jointly work toward recovery. In addition, it became clear that there are many unspoken tensions and complexities within the family, which interfered with the establishment of a well-functioning RG. Distance seemed to be accepted when there is conflict or disagreement, which reinforced Martin's (destructive) behavioral pattern. Thus, existing interactional difficulties stood in the way of establishing an open and honest alliance within the RG.

### Case 4: Raoul—The Struggle of Opening Up

When data collection begins, Raoul lives in a sheltered housing. He has a history of severe substance abuse and psychotic episodes, and he now wishes to be more independent from mental health care. In the first interview, he states that a psycho-education course 2 years ago taught him that the voices he had been hearing for about 20 years are actually his own. However, distinguishing them from reality takes a lot of his energy, and he is not able to do some kinds of work or daily activities. Raoul has nominated his mother, brother, and social worker to be part of the RG. He is enthusiastic and plays an active role in the setup of his RG. He borrows the case manager's book about the RG method, appoints himself the chairman of the RG meetings, and wants to take the lead in the in-depth preparatory conversations with the invited RG members. Nevertheless, Raoul indicates that he is not looking forward to the RG meeting because he does not like to be the center of the attention:

*Interview Raoul*. Raoul: “One hour […] That sounds so long to me, how are we going to fill one hour? […] and then I feel like, what do I have to say right now, why is it about me. Why do people find that important? So, it is difficult for me to express myself about myself.” […] Researcher: “So talking about yourself for an hour is difficult.” Raoul: I find it really troublesome, yes. I'm pretty much dreading it.”

At the start of the first RG meeting, Raoul asks the RG members to read the report of the in-depth preparatory conversation with his mother, explaining that everyone knowing about his past is a good start. From the report, it is clear that his mother has gone through a lot with Raoul. The past 10 years have been tough for her because she had to watch her son slip away while ceaselessly trying to save him. Despite the considerable improvement in their relationship since then, his mother repeatedly intervenes in the meeting with implicit references to the past. The researcher's field notes describe her clear need to be heard and persistent urge to share her struggles and fears with the professionals. Several times, she expresses that it is hard to have confidence in the future and support Raoul's wish to be more independent.

*RG meeting*. Mother: “He says that he wants to live independently, well then I just flinch, I take three steps back and…that is just a bitter pill to swallow. And I heartily wish it for him, but as he is now, I just really, really not see it happening.”

In response to the first RG meeting, the case manager encourages Raoul's mother to join a family psycho-educational program on psychosis and schizophrenia. At this program, she learns what her son's illness actually entails and how she can better relate to it. This changes the dynamics of the second meeting, and it stimulates her to reflect on the influence of her own behavior on Raoul's functioning:

*RG meeting*. Mother: “I wanted to push him, you know, ‘go for a walk, go for a nice run’. Well, you should definitely not do that. Because people who are schizophrenic seem to be really, really, really tired. Completely exhausted. So, at lesson 2 I already knew I shouldn't do that.” *Laughs*.

Despite the changed dynamics between Raoul and his mother, the second meeting has a tense atmosphere. In the period between the first and second meeting, Raoul had told the researcher that he is occasionally using drugs again. The mental health professionals know, but Raoul is terrified that his family will find out and demands that it will not be a topic during the RG meeting. The professionals respect his wish, although they struggle with the situation. In the period after the second meeting, they repeatedly confront him, expressing their own discomfort to address the subject of honesty and openness. Looking back at this period in an interview, Raoul says that although it was stressful at the time, the RG setting served as an incentive for self-reflection and confrontation of the situation. He decides to quit using drugs so that he will no longer have to lie to his family.

*Interview Raoul*. Raoul: “The RG has definitely accelerated that; that I have come to my conclusions, this is untenable, this cannot continue, it will go wrong somewhere. And also that I became aware of it; I just lied to her [mother], and that's really not okay. I couldn't pretend any more that I wasn't.”

This realization is a first step toward being honest and open with his family. After about 1 year of data collection, a similar event takes place. In consultation with the psychiatrist, Raoul decides to quit taking medication and involves his family in this decision. The RG meeting becomes a very honest conversation in which Raoul and his family open up and share their worries and fears with each other. After the meeting, Raoul tells the researcher that his family needs to feel that they are part of his decisions and that considering the perspectives and well-being of others gives him more gratification in the long term than making decisions by himself. In addition, he noticed that openness allows other people to come close, and that this had substantially improved his relations with both his family and the mental health professionals:

*Interview case manager*. Case manager: “[At first] he was absolutely inscrutable; I really had no idea what was going on inside him. And look at him now; yes, really it is a huge difference.”

In her interview, Raoul's mother explains that the increased openness is very important because it gives her confidence that she will not be left out again. Toward the end of data collection, Raoul, his mother, and his brother all state that the RG meetings have evolved into a place where they can be vulnerable, honest, and open with each other. Importantly, the topics of the RG meetings are no longer solely directed toward Raoul and his goals; they include the mutual relationships between Raoul and his family as well as the latter's vulnerabilities, fears, and behavior. Thus, their relationships become reciprocal, and the openness extends beyond Raoul's goals to cover broad aspects of daily life:

*Interview Raoul*. Raoul: “The last two times were just very open conversations, everything could come to the table and that gave me peace of mind and also my mother, I know that for sure. Apparently, we usually don't talk with each other so openly, and now the setting makes us ready to do just that. Yes, I found that a lot more pleasant.”

*Interview brother*. Brother: “I think the RG offers a stage to continue that [being vulnerable], as there is safety for everyone. And that the vulnerability does not only apply to Raoul, but also to us, as family. […] Yes, that certainly connects. Absolutely. That is, of course, what it is all about in a relationship: that you are honest with each other and that you share what is going on inside. That has been disturbed for a long time, and that it is now slowly repairing again; yes, that is really very valuable.”

#### Reflection

This story is characterized by increased openness in the communication between Raoul, his family, and the professionals. At the start, there was a pattern in which, based on past events, Raoul's family closely watched him and therefore exerted control out of fear. Raoul interpreted this as a lack of trust, which led him to keep things to himself. This, in turn, enhanced his family's fear. The RG meetings evolved into a place where this pattern was exposed and could be adjusted. The members all developed more self-reflective and vulnerable attitudes, and they gained an understanding of each other's past experiences. Raoul learned that being open to his family made them less suspicious, and he increasingly allowed them to be part of his decisions. This, in turn, increased his family's confidence and gave them the space to see him as a person with dreams and wishes instead of a patient they had to keep a close eye on. The mental health professionals contributed to this by not openly judging Raoul for withholding information from his family and instead repeatedly questioning the consequences and stimulating him to open up. Although it was a struggle for all members of the RG, these developments helped them jointly work toward opening up to each other and (re)building mutual trust. Remarkably, Raoul and his family indicated that they do not have these kinds of conversations in between the RG meetings; the fact that they are scheduled provided an opportunity to build equal, normalized relationships in which Raoul's illness was *not* the central topic. Hence, the RG meetings were a place where they could discuss the past and let issues go in daily life.

## Discussion

The RG method intends to promote patients' agency and self-management and organize collaborative partnerships between patients and their informal and formal support system. The present paper aimed to enhance the understanding of the interpersonal dynamics that arise within an RG as well as their influence on the recovery journey of the individual suffering from SMI. To this end, we narratively reconstructed the stories of four men—Leon, John, Martin, and Raoul—setting up RGs. Based on our analysis, below we explore the relations and interpretations of the unfolding processes within the four RGs, and we discuss possible implications for practice.

Within the RG method, patients are encouraged to be the director of their group and to take responsibility and ownership regarding their path to recovery (11–13). In the four stories, however, most of the RG members had long histories of dependence, risk prevention, and non-reciprocity with each other, and these existing interaction patterns—which varied in rigidness—interfered with the idea of agency of the patient. Thus, being the director of the group cannot be imposed; instead, a movement in the existing interactional patterns is needed to enable ownership and responsibility to emerge. The four stories illustrate how such interactional movements go hand in hand with struggles and interpersonal tensions.

For Leon and Raoul, being the director of their group led to pressure, fear of letting others down, and struggles with being fully open and vulnerable during the RG meetings. Leon tended to place himself below his significant others and thus take on the position of patient. For Raoul, his RG had trouble seeing him as a person with wishes and dreams instead of a patient on which they had to keep a close eye. For both, the process of moving beyond the role of patient and finding new balance in their relationships proved to be essential for establishing RGs that facilitate their empowerment. Importantly, this process required a shift in roles and restructuring of all RG members' perceptions of the relationships. In both stories, the RG meetings served as platforms for interpersonal patterns to be exposed and readjusted.

In the stories of John and Martin, no such shift in existing patterns was observed. John did not redefine his perception of agency and persisted in striving toward independence without help. The other RG members acted to protect him in order to reduce risks. Both John and his RG responded based on old patterns, and the RG meetings did not expose or help adjust them. The lack of change in interpersonal dynamics impeded John's recovery journey, as there was no room for him to take responsibility for both his strengths and weaknesses. Martin's RG process was too short to establish an actual group process. Martin and his family used distance and closeness to regulate their own feelings and regain control over the other. This interfered with the development of mutual trust and joint work toward recovery. Perhaps the expertise of an educated system or family therapist would have been helpful to explore the family's frustration with the mental health professional and increase their understanding of existing frictions and tensions. In this way, the first steps could have been taken toward cooperative partnerships, which could have served as a foundation for further work within the RG.

The analyses suggest that the degree to which the RG method contributes to recovery is strongly determined by the degree to which the existing roles of the patient and his/her RG members are changed. It is essential to break old, rigid patterns that are characterized by inequality and dependence. Jointly searching for a new balance in relationships is a vital process for establishing an RG that facilitates the patient's empowerment. Non-reciprocity can make individuals feel lonely, guilty, weak, incapable, indebted, and inferior, and such relationships, even when they provide much help, can be harmful to psychiatric clients in various ways (44). The stories of Leon, John, Martin, and Raoul show that breaking through old patterns is challenging. In addition, achieving social support within the involved relationships requires a delicate balance, as such support implies that a person is dependent on others, which tends to distance the helper from the person being helped (17, 44, 45). To change the mutual perceptions of relationships, it is essential to investigate the underlying emotions, fears, and attitudes of patients, their significant others, and the involved mental health professionals. An open and reflective atmosphere during the RG meetings stimulates members to explore and question their own roles, so working toward recovery goals becomes a shared and honest process.

The importance of openness and reflection for adjusting existing roles and patterns raises the question of how such an atmosphere within an RG arises or be facilitated. We saw that it can arise in response to an external event, such as the break-up between Leon and his partner, and that it can be stimulated by mental health professionals. When the professionals broadened their focus from Leon to the dialogue between Leon and the other RG members, the members started to reflect on themselves and the situation, and they became more direct and open toward each other and Leon. Similarly, when the professionals gave space to the concerns and fears of Raoul's mother, Raoul became more aware of the consequences of his behavior on his family, and the communication between them became more open and honest. Thus, it is important that all RG members are invited to play an active role and to consider what they truly need to believe in the goals and participate in achieving them.

By recognizing the importance of including the social context in understanding, analyzing, and responding to mental health difficulties and recovery (17, 45, 46), the RG method is best be viewed as a person- and network-oriented approach. Indeed, our findings are in line with identified working mechanisms of meaningful and sustained inclusion of the social network. These have been found to be characterized by collaboration principles, which promote deep listening to the lived experience of families; a commitment to work in equal partnership with service users and family members; an openness to acknowledge, articulate and address power relations; and a commitment to change service delivery cultures (47–50). Above all, such approaches firmly recognize that no one exists in isolation. In contrast, most people's lives are defined by their networks and relationships, and problems and solutions are socially constructed through shared language and understandings (51).

An influential example of such approach is Open Dialogue (OD) (52, 53). The approach aspires to create a space where decision making is transparent and service users are able to find new words for their experiences. Studies of OD can be helpful in further developing and shaping the RG method. Mechanisms of change in OD have been identified (54, 55) and seven key elements were outlined in fidelity criteria (56). These elements can be understood as related to both the organization of services and a way of being with people, the latter including the elements of tolerating uncertainty and dialogism (57). Future studies should investigate their similarities, differences and lessons to learn to establish the social and contextual nature of recovery in treatment and care for people with SMI.

### Clinical Implications

Mental health professionals' role is to monitor the processes within the RG by inviting RG members to share their thoughts and feelings; stimulating openness about frictions or differences in point of view; acknowledging and investigating the positions and needs of patients' significant others; and provoking curiosity of each RG member about themselves, the situation, and the group process. This stimulates members to re-think their roles, needs, and behaviors (17, 50). The stories of Leon and Raoul show that this not only facilitates openness but also increases mutual understanding. If individuals feel that they are understood by someone, they will be inclined to learn from them (58). Hereby, the RG serves as a “we,” and as a collaborative learning community in which new knowledge and meaning arise from mutually influencing processes (57, 59). The functioning of the social network as a whole and the ability of the involved members to respond in different ways are important when working toward the patient's recovery goals.

By making space for all RG members to be heard, the RG itself and the RG meetings could evolve into a *holding environment*, a safe setting that enables individuals to explore new methods of interaction and communication (60). The holding environment can serve as a safe place in which people in recovery and their significant others feel that they can take risks, consider each other's perspectives, and explore their true feelings (61). The professional is part of this holding environment and thus is an equal partner in the process, as opposed to an expert that brings knowledge (62–65).

Cultivating such attitude and taking on a monitoring role within the RG involves a subtle but significant shift in the dynamics between mental health professionals and patients and their significant others and is reshaped to “doing *with*, rather than doing to and doing for” (66). Developing appropriate skills is not restricted to a certain professional background but training and supervision is recommended [see (8, 14, 31)].

### Methodological Considerations and Limitations

First, the uniqueness of the recovery journeys of the participants and the small sample size limits the generalizability of our findings to a wide population of people with SMI. The findings of this study are rooted in time, place, and person and future studies should investigate the role of specific characteristics, such as illness acuity, ability of self-reflection, and different phases of illness on group dynamics for further application of the RG method. Above all, the paper is meant to stimulate reflection and thinking about the different ways the RG method takes shape in clinical practice. Hereby, we hope that our analysis encourages mental health professionals to embrace the uniqueness of each individual RG and adapt to the personal needs of its involved members.

Second, hermeneutical analysis is based on the idea that data cannot be regarded as purely isolated information units that can be observed separately by other researchers. Rather than trying to eliminate the effects of the researcher, researchers should try to understand and exploit them (67). Therefore, continuous reflexivity regarding our impact on the data, analysis, and interpretations was important throughout all phases of the study. To that end, the first and second author were in continuous dialogue with each other to ensure they remained open and curious about the participants' unique situations. During data collection, they critically questioned each other to gain an understanding of the origin of certain beliefs and interpretations that could affect the course of the interviews. During data analysis, the first and second author jointly reviewed all transcripts and field notes, made memos of their discussions, and eventually achieved intersubjective agreement on their interpretations. It is thus important to take into account, when reading the paper and interpreting the analysis, that their personal and professional experience and knowledge inspired and informed the analysis and interpretations (68).

Third, the confidential relationships between the first author and the participants (both patients and significant others) were important in the interpretation process. The first author followed the four stories for a longer period of time and attended all RG meetings. Participants shared deeply personal information and vulnerabilities throughout the process, which indicates that they saw the researcher as a trusted partner. Initially derived meanings and hypotheses regarding the participant's recovery process and the interpersonal dynamics within the RG were discussed with the participants to jointly interpret the data. This was one of the main strengths of the study as the research became an equal and joint exploration and investigation. At the same time, the attention and sincere interest for the participants and the repeated visits might have had a therapeutic influence that may have been tangled with the method. In addition, the researchers repeatedly asked to evaluate and reflect on the RG method and its influence on the recovery journey, which may have led to an attributed importance of the method for the participants, that would otherwise not have been experienced or interpreted that way.

## Conclusions

Taken together, by reconstructing the four stories, we aimed to gain insight into the different ways the RG method takes shape in the four men's lives. The stories showed that the RG method should not be considered an intervention for organizing informal support for the “designated” patient, but as a platform for changing the functioning and dynamics of the social network as a whole. For a well-functioning RG, it seems essential to break through old patterns of inequality and dependence and work toward openness and reciprocity in interpersonal dynamics. The four cases showed that it takes time, patience, and small steps back and forth to jointly overcome the struggles and fears related to finding new ways of relating to each other. An honest and reflective atmosphere in which all participants are encouraged to participate and be curious about themselves and each other is essential for changes in interpersonal dynamics to emerge. Such changes pave the way for individuals with SMI to find their own voices and pursue their unique recovery journeys.

## Data Availability Statement

The datasets generated for this study are available upon request to the corresponding author.

## Ethics Statement

The studies involving human participants were reviewed and approved by Medical Ethical Committee VU Medical Centre. The patients/participants provided their written informed consent to participate in this study.

## Author Contributions

CT and JB were responsible for data collection, performed the interviews and analysis, and wrote the first draft of the paper. CM and HK were involved in the several rounds of analyses and provided comments on drafts of the paper. All authors contributed to the study design and concept development.

## Conflict of Interest

The authors declare that the research was conducted in the absence of any commercial or financial relationships that could be construed as a potential conflict of interest.
